# Evaluating the Impact of Intimate Partner Violence: A Comparison of Men in Treatment and Their (Ex-) Partners Accounts

**DOI:** 10.3390/ijerph18115859

**Published:** 2021-05-29

**Authors:** Berta Vall, Anna Sala-Bubaré, Marianne Hester, Alessandra Pauncz

**Affiliations:** 1 European Network for the Work with Perpetrators (WWP EN), 12681 Berlin, Germany; alessandra.pauncz@work-with-perpetrators.eu; 2 Faculty of Psychology, Educational and Sports Sciences, FPCEE, Ramon Llull University, 08022 Barcelona, Spain; annasb4@blanquerna.url.edu; 3 School for Policy Studies, University of Bristol, Bristol BS8 1TZ, UK; Marianne.Hester@bristol.ac.uk

**Keywords:** intimate partner violence, impact, perpetrator programmes, victims’ safety, negative consequences, psychological consequences, health consequences

## Abstract

Intimate partner violence (IPV) is a public health and widespread problem, and perpetrator programmes are in a unique position to work towards the end of gender-based violence. However, in order to promote safe perpetrator work, it is crucial to focus on the impact of IPV on the victims and survivors. In this context, little research has triangulated data by including both, victim’s perspectives on the impact that IPV has on them and also men’s level of awareness of the impact of their violent behaviour. In this paper, results from the “Impact Outcome Monitoring Toolkit (Impact Toolkit)” from one perpetrator treatment programme in the UK are presented. Participants were 98 in total; 49 men that were following treatment in a perpetrator program and their (ex-) partners. The differences in their perceptions of the IPV, but also on the impact of this abusive behavior on the victims, is described. Finally, recommendations for research and practice are discussed.

## 1. Introduction

Intimate Partner Violence (IPV) is defined by the World Health Organisation (WHO) as “any behaviour within an intimate relationship that causes physical, psychological or sexual harm to those in the relationship” [1] (p. 1). IPV is a public health [2,3,4] and widespread problem; affecting nearly 1 in 4 women worldwide —including data from both the USA [5] and Europe [6]. Whereas men are more likely to experience violent acts by strangers or acquaintances; the most common perpetrators of violence against women are male intimate partners or ex-partners [1], and thus, women are most likely to report violence by an intimate partner than by any other perpetrator [7]. Therefore, women are much more likely to suffer major impacts and consequences of IPV compared with men, such as being injured or killed by an intimate partner [8].

In order to promote safe IPV perpetrator work, it is crucial to consider not just the presence and frequency of the abusive behavior but also the impact of this behavior on the victims and survivors. Research on the impact of violence has found that even if the short-term psychological consequences might be similar for partner and non-partner physical and sexual violence, the long-term psychological consequences are greater when the violence comes from a partner than when it is from a non-partner, emphasizing the consequences of a pattern of repeat victimization in IPV [6]. Previous research has focused on both physical and mental health consequences. The main physical health outcomes from IPV refer to (a) short-term and/or direct physical impacts such as homicide, and physical injuries [9]; and (b) long-term and/or indirect physical impacts such as traumatic brain injury, memory loss, adverse pregnancy outcomes, chronic pain syndromes, among others. The main psychological impacts referred by the victims/survivors themselves are (a) short-term psychological impacts, such as anger, fear, or shock; and (b) long-term psychological impacts, such as anxiety, loss of self-confidence, or feeling vulnerable [5]. Regrettably, the FRA survey did not analyze the impact of psychological IPV, despite this, several studies have pointed out that psychological abuse has at least as important consequences as physical abuse [10,11,12]. 

IPV is understood and lived very differently from the perpetrators’ and victims’ side. Thus, their accounts might differ much when understanding or explaining the IPV situation that has happened. Even in terms of the attributions given for an abusive behavior some differences appear and victims give different explanations of why perpetrators exerted a given behavior than the ones’ given by the perpetrators’ themselves [13]. In the context of evaluating the prevalence and impact of IPV, including triangulation of data is a crucial aspect [14]. It is important to analyze the perception that each member of the couple has about the abusive behaviour, especially at the beginning of the intervention programme but also throughout the course of the treatment. 

Few studies have analyzed the perpetrators and their (ex-) partners accounts on the prevalence and/or impact of the violence behavior. One of the first researches that compared abusive men accounts with the ones of their (ex-) partners found that for physical abusive behavior men generally reported less violence (prevalence) and that this difference was even more pronounced when comparing the reports on the frequency of those violent acts. As for the consequences of violence, women consistently reported receiving more injuries and more frequently than the ones reported by men. For the controlling behavior there was more concordance on the prevalence but not on the frequencies, being the women who reported more frequency of these behaviors [15]. Sadly, this study did not analyze the consequences of the controlling behavior, moreover psychological consequences of violence (either physical or psychological) were not considered. 

In another study, Gondolf conducted a four-year follow-up evaluation of a perpetrator IPV programme in the US and found that the main predictor of re-assault was the women’s perception of their safety and concern of being re-assaulted [16]. Thus, contacting the partner of IPV perpetrators is essential, not only for the purpose of ensuring the partner’s safety, but also for evaluating the progress of the men in treatment [17]. Moreover, as suggested in the Mirabal project, the measurement of the consequences and/or impacts of the violent behavior should include, not just a reduction of its impacts stated by women but also a measurement of the men’s level of awareness of the impact of their violent behavior on women and children as an important measure of success [18]. This is a crucial aspect in order to assess if men that enter the treatment are aware of those impacts and how this awareness plays a role in the final treatment outcome. To date, one of the few studies that has analyzed this aspect, the Mirabal project, found that according to both men in treatment and their (ex-) partners, there was an increased understanding of the impact of his abusive/violent behavior. However, the Mirabal project used different measurement tools for men and their (ex-) partners to assess their perceptions/reports of violence and of the impact of this violence, which hinders comparison between them. This is the first study in which men and (ex-) partners accounts around the presence, frequency and impact of the violent/abusive behaviour (including both short-term and long-term psychological and physical impacts) were compared using the same tool for both, and some interesting results have been obtained. Using the same tools to assess both perpetrator’s and (ex-) partners perceptions of violent/abusive behavior, and more specifically, its presence and frequency and its impacts, is crucial to learn the ways violence is perceived within couples, which in turn can guide more tailored and targeted treatment programmes. 

All in all, in this paper we aimed at answering the following questions: (1) are the men in treatment and their (ex-) partners views on the violent/abusive behavior similar or different at the beginning of the treatment and on which specific aspects do they converge or differ (frequency of violent/abusive behaviors, presence, type etc.)? and (b) is the impact of the violent/abusive behavior perceived differently when comparing the men in treatment accounts with the ones’ of their (ex-) partners) at the beginning of the treatment? The tool used, the Impact Outcome Monitoring Toolkit (Impact Toolkit in what follows), allows the gathering of the same information from both parties, and thus to make comparisons within groups and within couples. 

## 2. Materials and Methods

### 2.1. Participants and Setting

Participants in this study were 49 male perpetrators of IPV enrolled in the Abusive Behavioural Change (ABC) Project for male and female perpetrators of domestic abuse, and their (ex-) partners. This programme was run by the Elm Foundation in Chesterfield, in the UK. The programme is part of a multi-agency approach against domestic violence, and regularly collaborates with police, and different social services; thus, participants are usually referred by social and child protection services or are self-referred. 

The 49 (ex-)couples were all the couples that joined the ABC programme between January 2017 and July 2019, and in which both the men and their (ex-)partner completed the Impact Toolkit at the beginning of the programme. They were from different cohorts of the ABC project, but the main characteristics of the programme and the requirements and profile of eligible participants have remained fairly stable. To participate in the programme, men were required to demonstrate at least some motivation prior to participation in the programme, and have to agree not to misuse alcohol and use drugs during the program. Participants were from a wide range of ages (see Table 1), with the majority between the ages of 22 and 50. None of them had severe mental disorders or cognitive impairment.

According to the men, 16 of these couples were no longer in a relationship. The remaining were either living apart (*n* = 18) or together (*n* = 11) and four were unsure about the status of their relationship. Most of them had children (*n* = 40). They were typically employed full-time (*n* = 32), while some were unemployed or unable to work (*n* = 12) and the rest were either employed part-time, studying or a combination of the former (*n* = 5). Reflecting the range of employment activity, participants were also diverse regarding their socioeconomical status (see Table 2).

Men were referred via a variety of channels. A high number of men were being pressured to attend by child protection and the social services regarding access to children (*n* = 25), which was the most common referral route to the programme, involving more than half of the men who had children. Other channels were self-referral following publicity (*n* = 5), referral via mental health services (*n* = 5), police (*n* = 3), civil court mandation (*n* = 3), helpline (*n* = 3), (ex)partner (*n* = 2), health services (*n* = 2), relationship counselling service (*n* = 2), family and friends (*n* = 1), probation (*n* = 1), and solicitor (*n* = 1).

### 2.2. Instrument: The Impact Toolkit Questionnaire

The instrument used in this study was the “Client and (ex)partner Impact Questionnaire”. This questionnaire is part of the Impact Outcome Monitoring Toolkit of the “European Network for the Work with Perpetrators of Domestic Violence (WWP EN)”. The toolkit comprises eight versions of the questionnaire, slightly adapted in relation to the phase (four versions: T1-beginning of programme, T2-in the middle, T3-at the end of the programme, and T4-follow-up) and in relation to the respondent (two versions: perpetrator and -ex-partner).

In line with this paper’s aim to compare men’s and (ex-) partners’ perceptions of IPV at the beginning of a training programme, in this study we focus on the responses to the questionnaire for perpetrators and (ex-) partners at T1 (beginning of the programme), and on two scales of the questionnaire: violent/abusive behaviour and impact of violence/abuse. All the items of the two scales are equivalent across the men’s and (ex-) partners’ questionnaires. The first scale contains 29 items divided into three sub-scales regarding three types of IPV: emotional (10), physical (12) and sexual behaviour (7). These sub-scales can be evaluated at three different levels of frequency (never, sometimes, often). The second scale, Impact of violence, comprises 16 items. It contains items about physical and emotional impacts on the (ex-) partner. Finally, the questionnaire also included three questions regarding the impact of IPV on children, related to whether the respondent thought children were affected by the abuse and whether they thought children were angry with the men and with the partner.

### 2.3. Data Collection

Responses from the men and (ex-) partners were collected at the beginning of each round of the programme. The procedure used to collect the answers was different for each group. Men responded to the questionnaire on-site and on paper. They did it alone, but a facilitator was present in the room to assist with any questions or clarifications they might have. 

Partners and ex-partners are contacted at the beginning of the programme to inform them about the programme, its content and methods, the support services in case they needed them, and to learn about their experience of violence and their assessment of the effectivity of the programme. Thus (ex-) partners responded to the questionnaire as part of the protocol of (ex-) partners’ contact with the women’s support service. Responses were collected either over the phone or face-to-face depending on the involvement and availability of each person.

### 2.4. Variables

The present study compared men and (ex-) partners perceptions regarding IPV in relation to the following groups of variables. Each of these variables was calculated for each individual in both groups following the same procedure:

- Frequency of IPV. Six variables: three variables for each group for each type of abusive behaviour: emotional, physical, and sexual abusive behaviours, for each group of participants. Means were calculated for each sub-scale of the violent/abusive behaviour scale.
- Number of IPV behaviours. Six variables: three variables for each group for each type of abusive behaviour: emotional, physical, and sexual abusive behaviours, for each group of participants. Each item of the three sub-scales of the violent/abusive behaviour scale was recoded into a new binary item identifying presence (1) or absence (0) of each behaviour. The total number of present behaviours was calculated for each of the three sub-scales.
- Presence of IPV. Six variables: three variables for each group for each type of abusive behaviour: emotional, physical, and sexual abusive behaviours, for each group of participants. These three variables were binary variables that identified the presence or absence of abusive behaviours of each type. The number of IPV behaviours for each sub-scale (see above) was recoded into presence (1) or absence (0) of each type of behaviour.
- Impact of IPV on (ex-) partners. The number of impacts reported in the impact of violent behaviour scale was only counted by individual. The same analyses were conducted for (ex-) partners’ answers.
- Impact of IPV on children. Three variables: direct responses to the questions regarding impact of IPV on children were included.

### 2.5. Analyses

As we were interested in any differences between men and (ex-) partners within each couple, not in the differences between the two groups in general, responses from men were paired with data from their (ex-) partners. Thus, data were introduced into the database by couple and not by individual. Each case of the database (*n* = 49) contained the answers of the man paired with those of his (ex-)partner.

**Figure 1:**
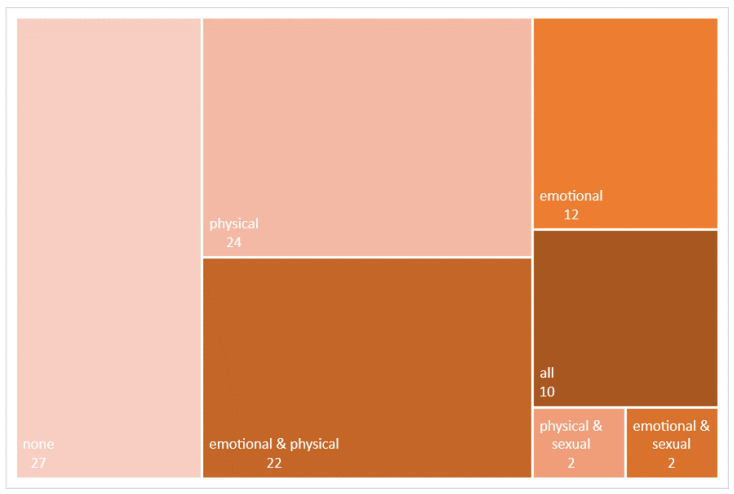
Percentages of each profile according to the type of violent behaviour they reported they had exerted.

**Figure 2:**
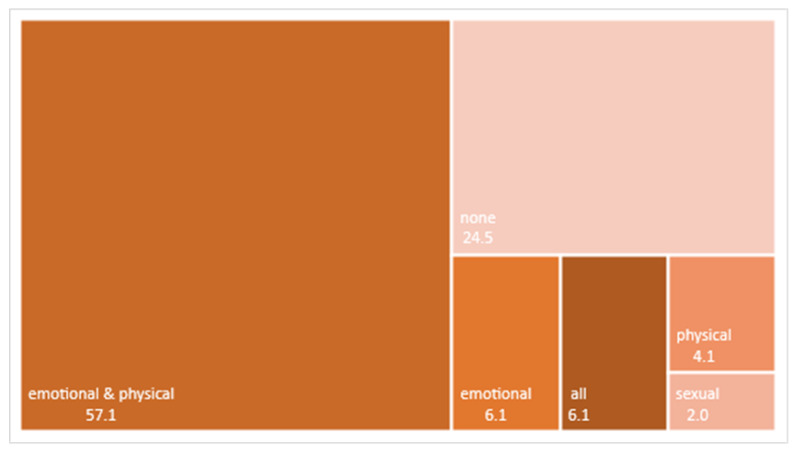
Percentages of profiles of (ex-) partners according to the type of violent behaviour they reported they had suffered.

Descriptive statistics (mean and frequencies) for each variable and group were calculated. All the items in the two scales were given the same relative importance. We assigned a ‘type of violence profile’ to each man and (ex-) partner using the Presence of IPV variables (i.e., presence of emotional, physical, and sexual abusive behaviours). By combining these three variables, eight possible profiles emerge (none, only emotional violence, only physical violence, only sexual violence, emotional and physical violence, emotional and sexual violence, physical and sexual violence, and all three types of violence). Each individual was assigned to one of these profiles based on the type(s) of violence they reported. Frequencies of each type of profile were calculated and graphically displayed in Figure 1 and Figure 2.

Men and (ex-) partners’ perceptions about the frequency and number of emotional, physical and sexual abusive behaviours were compared using Wilcoxon signed-rank test, the non-parametric test used to compare matched samples. The Wilcoxon test was also used to assess differences in the impacts of violent behaviour on (ex-) partners both for the total number of impacts and for each item of the scale, and to compare men and (ex-) partners in regard to the impact of IPV on their children. Bivariate correlations among the measures of frequency of IPV behaviours (6 variables; 3 for men and 3 for (ex-) partners) and among measures of number of IPV behaviours (6 variables; 3 for men and 3 for (ex-) partners) were calculated using Pearson’s correlation coefficient. 

### 2.6. Ethical Approval

At the beginning of the programme, participants are required to sign a commitment contract, which also includes a data protection agreement informing them about the use and treatment of their personal information. All the participants agreed that their data would be used only for therapeutic and research purposes and that any use of this data for research purposes would ensure, at any time, the confidentiality and complete anonymity of data. Men and (ex-) partners were assigned an identification number which was used to answer the questionnaire. Programme staff were responsible for individuals’ personal information and the identification number assigned to each person. The authors of this paper did not have access to participants’ personal information at any time of the research.

## 3. Results

### 3.1. Presence of Violent Behaviours

At the beginning of the programme 23 men reported some kind of emotionally abusive behavior, 29 admitted at least one physical abusive behaviour and only 7 reported at least one sexual abusive behavior. In contrast, 34 (ex-) partners reported suffering some kind of abusive behavior, 33 reported at least one physical abusive behavior and only 4 reported suffering sexual abusive behavior. In the within pairs analysis, these differences were only statistically significant for presence of emotional abusive behaviour (*p* < 0.01).

The combination of the presence or absence of the three types of abusive behaviour yielded different profiles of violence among men, illustrated in Figure 1. The most frequent profiles were men who did not report any type of behaviour (27%), those who only reported physical behaviours (24%), and those who reported emotional and physical behaviours (22%). The other combinations were less frequent, with 10% of men reporting the three types of behaviour, and none reporting only sexual behaviours.

Figure 2 shows the distribution of the profiles of violence among (ex-) partners. In contrast to men’s profile, the most frequent profile by far were (ex-) partners who reported emotional and physical behaviours (57.1%), followed by those who did not report any type of behaviour (24.5%). The other combinations were less frequent, with only 6.1% of (ex-) partners reporting the three types of behaviours, and none reporting the combination physical + sexual behaviours and emotional + sexual behaviours.

When comparing the profile report by men and their (ex-) partners within each couple, results show that less than a third of them (*n* = 15) coincided in the profile they reported: six reported no violent behavior of any type, eight reported emotional and physical but no sexual violent behaviors; and in one (ex-) couple both parties reported the three types of violent behavior. In the other 34 (ex-) couples, man and (ex-) partner reported different violent behavior profiles. The most prevalent combinations among them were partners who reported both emotional and physical violent behaviors with men who reported only physical behavior (*n* = 6), only emotional behavior (*n* = 5), all three types of behavior (*n* = 4) or no behaviors at all (*n* = 3).

### 3.2. Frequency and Number of Violent Behaviours

The mean frequency of violent behaviour, from the perspective of the men, was very low for the three scales (an average of 1 would indicate complete lack of violent behaviour), especially for sexual behaviour, with no differences between emotional and physical behaviours in terms of frequency (see Table 3). Regarding the number, they admitted slightly more physical than emotional behaviours, and the average for sexual behaviours was close to zero.

(Ex-) Partners considered violent behaviours to be slightly more frequent than men reported, and they reported experiencing a bigger number of violent emotional and physical behaviours than men did. The Wilcoxon test for paired samples showed these differences to be significant for frequency and number of emotional behaviours (*p* < 0.01) and for frequency and number of physical behaviour (*p* < 0.05). Men and (ex-) partners were not different in regards to both measures of sexual behaviour.

Within the group of men, the most frequently mentioned emotional behaviours were “Told partner what to do/not do, where to go/not go, who to see/not see” (*n* = 15); “Extreme jealousy or possessiveness” (*n* = 15); and “Threats to hurt partner/ex” (*n* = 12). As for the physical behaviours, the most frequent behaviours, according to men, were: “Slapped / pushed / shoved” (*n* = 23); “Restrained/held down/tied up” (*n* = 15); and “Physically threatened” (*n* = 13). Finally, the most frequently mentioned sexual behaviours were: “Disrespected boundaries or safe words” (*n* = 4); “Touched in way which caused her fear/alarm/distress” (*n* = 4).

According to (ex-) partners, the most frequent emotional behaviours were “Extreme jealousy or possessiveness” (*n* = 28); “Told what to do/not do, where to go/not go, who to see/not see” (*n* = 22); and “Threats to hurt you” (*n* = 21). As for the physical behaviours, the most frequent behaviours, according to (ex-) partners, were: “Slapped / pushed / shoved” (*n* = 26); “Stalked/followed/harassed you” (*n* = 18); and “Restrained/held down/tied up” (*n* = 17). Finally, the most frequently mentioned sexual behaviours was: “Touched in way which caused fear/alarm/distress” (*n* = 4).

### 3.3. Relationship among Types of Behaviours Reported by Men and (Ex-) Partners

Correlations among frequency and number of all violent behaviours for both men and (ex-) partners yielded statistically significant correlations among all eight measures of emotional and physical behaviours (frequency and number according to men and (ex-) partners) (see Table 4 and Table 5). This shows not only that there is a relationship between emotional and physical behaviours but also that this relationship is still very significant across groups (e.g., the higher number of emotional behaviours reported by a client, the higher number of physical behaviours reported by his ex-/partner), which in turn also shows agreement between men and their (ex-) partners in all these measures. However, none of the measures of emotional and physical behaviour correlate significantly with any of the measures of sexual behaviour. In this scale, there are no significant correlations between measures of men and (ex-) partners either, showing a lack of agreement between the two groups. However, it must be noted that sexual behaviour was very rare in this group, according to men and (ex-) partners, which might, at least partially, account for the lack of significance.

### 3.4. Impact of Men’s Behaviour on (Ex-) Partners and Children

Table 6 shows the frequencies of the number of impacts reported by individuals in each group (note the descriptive analysis here is done by separate groups and not by pairs). As seen, there was great variety in the number of impacts reported by men and their (ex-) partners men M = 6.18, SD = 4.79; (ex-) partners M = 7.39, SD = 4.99). Within the pairs, Wilcoxon test for matched samples showed there were not statistically significant differences (*p* = 0.094).

Looking at the individual items, Table 7 shows the frequencies of each type of impact according to each group (note here too the descriptive analysis was conducted separately for each group). For men, the most frequent impacts on their (ex-) partners were “sadness”, “anger/shock” and “loss of respect for them”, whereas for (ex-) partners, the most frequent impacts were “sadness” and “loss of trust”, but also “wanting to leave men”, “anxiety/ panic/ loss of concentration”, and “injuries such as bruises, scratches or minor cuts”. Frequency was only higher among men than (ex-) partners for “sadness” and “loss of respect”, and the rest of items had higher frequencies among (ex-) partners. 

Within pairs, Wilcoxon test for matched samples showed statistically significant differences between men and (ex-) partners in regards to three items: “Injuries such as bruises/ scratches/ minor cuts”; “Depression or sleeping problems”; and “Partner having to be careful of what she said or did”. (Ex-) partners reported all these items more frequently than men.

Finally, as for the impact of men’s behaviour on children, among the 40 men who had children, most of them thought the children were affected by the abuse and only 3 thought they were not. Although this number is higher according to (ex-) partners (n = 8), the differences are not statistically significant. The number of men and (ex-) partners who thought the children was upset with the men or the partner were very similar. 

## 4. Discussion

This is the first study in which men and (ex-) partners accounts around the presence, frequency and impact of the violent/abusive behaviour were compared using the same tool for both, and some interesting results have been obtained.

In regard to the presence and frequency of abusive behaviours several issues have emerged. First, comparing the most common emotional, physical, and sexual abusive behaviours stated by the men and their (ex-) partners, they seemed to be quite similar. The main difference was with regard to the “stalking” that (ex-) partners mentioned as one of the main abusive behaviours, whereas the men did not recognize it. 

Second, despite having quite similar views on the most common abusive behaviours, there were differences between the men and their (ex-) partners regarding the number and frequency of the emotional abusive behaviours. This was also visible in the profiles of violence per individual: while many men reported only physical behaviour, this profile was very rare among (ex-) partners who mostly reported physical connected to emotional behaviours. This seems to indicate that some men struggle to recognise or admit emotional abuse, which resonates with previous studies which found that the higher presence of emotional abuse, the greater possibility that victims also experience physical abuse [5]. Moreover, psychological abuse seems to be not just difficult to identify or recognized by men in treatment, but it also seems to be more pervasive as previous studies identified that psychological violence has the lowest rates of reduction in perpetrator programmes [6,16,17]. 

Third, despite those differences, correlations among these two types of behaviours are still significant, which indicates that, although men report fewer emotional behaviours, the amount and frequency of these behaviours they report is similar to their (ex-) partners’ reports; and that the more emotional behaviours they report, the more likely they are to report more physical behaviours (both men in treatment and their (ex-) partners). 

This study has shown that the reports by men in treatment and their (ex-) partners are correlated, and so they seem to be able to identify an increase and/or changes in the abusive behaviour in a similar way. Despite this, the total amount and frequency of abusive behaviours detected by both is still quite different, and so, in order to assess risk and safety, it is crucial to always count on (ex-) partners’ accounts. As already pointed out by Gondolf [16], the (ex-) partners’ accounts are the most reliable source for predicting re-assault. 

Fourth, sexual abusive behaviours followed a completely different pattern in all the measures. Although there were no differences between the men and their (ex-) partners with regard to the frequency and number of sexual behaviours reported, when the profiles of each individual are analysed, surprisingly, more men report exerting sexual abusive behaviour. Moreover, sexual abusive behaviour did not correlate with the other violent behaviours, and there was no correlation between men’s and (ex-) partners’ perceptions of this type of abusive behaviour either. The low number of reported behaviours of this type could partially explain these results. Another plausible explanation would be that there is a general reluctance by both perpetrators and victims to disclose sexual violence; as showed in several reports, disclosure of adult sexual violence is quite low [19,20]. Indeed, sexual assaults are less likely to be reported that physical assaults, while assault by a current partner are the least likely to be reported of all, even when compared to other types of sexual assaults [21]. When exploring the reasons for this low disclosure rate, it has been found that the fact that victims think it was a trivial abuse, thus minimizing its importance, was the main reason, followed by feelings of shame/embarrassment, and self-blaming [20]. Moreover, perpetrators in the criminal justice system who have carried out sexual violence (including in IPV context) mostly deny this (e.g., [22]). 

Finally, a significant number of men, but also (ex-) partners, did not acknowledge that the men used any emotional, physical and/or sexual abusive behaviours. This could be the result of a series of factors: first of all, possible denial of violence from the perpetrators side (especially when filling the surveys on their own), and also some minimization of violence from the victims side [23] as a result of living in a relationship with high prevalence of violence. Finally, the fact that the survey asked for some specific behaviours might have left out some of the abusive behaviours that were occurring within the relationship.

Regarding the impact of men’s violent behaviour, the main results obtained show, first, that both groups reported a similar number of impacts, but the most reported impacts were different; therefore, it seems that men recognize a similar number of impacts than their (ex-) partners, but those impacts are slightly different than the ones recognized by the (ex-) partners. More specifically, men had more difficulties identifying their (ex-) partners’ short-term physical impacts (such as bruises/ scratches/ minor cuts), and long-term psychological impacts such as: depression or sleeping problem; and the fact that the partner had to be careful of what she said or did because of the long-term coercive control exerted. 

Finally, regarding the impact of the abusive behaviour on the children, most of men are aware of the impact that the abusive behaviour had on them, which might be influenced by the fact that many of them men in this study had been told by social services/child protection to attend the programme. 

In what refers to the limitations of this study first of all it must be considered that the findings of this study are specific of a particular context, time, and group of individuals, and might be not representative of men and their (ex-) partners in other regions, with different backgrounds and cultures. Particularly, too, comparisons within couples with higher levels of violence might also yield different but very interesting and necessary insights. Moreover, it must be bear in mind that it was not possible to obtain an explanation for the reason of the differences observed among men in treatment and their (ex-) partners within this study as we did not include any open questions that would have allowed for a qualitative analysis of these reasons. For future studies, we aim at including qualitative data in order to explore further explanations. Moreover, in future research it would also be interesting to include information on the changes that men did during the course of the treatment, and on the evolution of these couples during the treatment. Finally, as recommendations for future research, it could be interesting to undertake an in-depth analysis of the profiles of violence according to men and their (ex-) partners including information of history of abuse or exposure to IPV in childhood for both participants. 

## 5. Conclusions

Comparing the accounts of men in treatment and their (ex-) partners has shown interesting results and has given some indications that are crucial for perpetrator programmes. It has become clear that in order to ensure safety and to assess the risk of perpetrator programmes it is crucial to include the (ex-) partners’ accounts as their accounts on the frequency and number of violent/abusive behaviors differ from the ones of their partners (i.e., men in treatment). Moreover, the impact of this violent/abusive behavior is also seen differently from both members of the couple, therefore, having the (ex-) partners’ accounts might help on increasing awareness (or accountability) of these impacts on the men’s side. All in all, the results obtained in this study have clear implications on perpetrator programme practice, and support the survivor contact element of IPV perpetrator programmes. As suggested by previous studies [24] the survivor contact element of IPV perpetrator programmes is mostly seen by survivors in a positive way (they felt validated, supported, learned about abusive behaviors, etc.). The present study supports this procedure as a very valuable way to evaluate the programme process and outcome.

## Figures and Tables

**Table 1:** Age group of male perpetrators.

Age	Freq	%
18–21	1	2.0
22–30	19	39.2
31–40	11	23.5
41–50	14	27.5
51–60	2	3.9
over 60	2	3.9
Total	49	100

**Table 2:** Socioeconomical status of participants in this study.

Status	Freq	%
Struggling essentials	5	9.8
Managing essentials, no left over	10	21.6
Occasional treat or save	20	41.2
Regular treats and saving	4	7.8
Comfortably managing	10	19.6
Total	49	100

**Table 3:** Frequency and number of violent behaviours reported by men and (ex-) partners.

Type of Violent/Abusive Behaviour	Men				(Ex-) Partners			
	Frequency		Number		Frequency		Number	
	M	SD	M	SD	M	SD	M	SD
Emotional abusive behaviours	1.19	0.25	1.57	2.11	1.46	0.52	3.22	3.22
Physical abusive behaviours	1.15	0.17	1.49	1.71	1.26	0.31	2.49	2.6
Sexual abusive behaviours	1.03	0.07	0.18	0.49	1.05	0.19	0.29	1.17

**Table 4:** Correlations among the frequency of the three types of violent behaviours of both groups.

Type of Violent /Abusive Behaviour	Physical Men	Sexual Men	Emotional (Ex-) Partners	Physical (Ex-) Partners	Sexual (Ex-) Partners
Emotional Men	0.62 **	0.17	0.45 **	0.52 **	0.01
Physical Men		0.10	0.42 **	0.59 **	−0.16
Sexual Men			0.13	−0.07	−0.03
Emotional (ex-) partners				0.75 **	0.16
Physical (ex-) partners					−0.04

** *p* < 0.01.

**Table 5:** Correlations among the number of the three types of violent behaviours of both groups.

Type of Violent /Abusive Behaviour	Physical Men	Sexual Men	Emotional (Ex-) Partners	Physical (Ex-) Partners	Sexual (Ex-) Partners
Emotional Men	0.50 **	0.20	0.41 **	0.55 **	0.00
Physical Men		0.17	0.31 *	0.46 **	−0.15
Sexual Men			0.19	−0.02	−0.06
Emotional (ex-) partners				0.72 **	0.17
Physical (ex-) partners					−0.06

* *p* < 0.05, ** *p* < 0.01.

**Table 6:** Number of impacts reported by men and (ex-) partners.

Number of Impacts	Men		(Ex-) Partners	
	Freq	%	Freq	%
0	8	16.3	5	10.6
1	2	4.1	2	4.3
2	4	8.2	1	2.1
3	2	4.1	2	4.3
4	4	8.2	5	10.6
5	8	16.3	4	8.5
6	1	2.0	3	6.4
7	3	6.1	1	2.1
8	0	0	1	2.1
9	2	4.1	1	2.1
10	1	2.0	3	6.4
11	3	6.1	5	10.6
12	4	8.2	5	10.6
13	4	8.2	2	4.3
14	2	4.1	6	12.8
15	1	2.0	1	2.1
Total	49	100	47 *	100

* Two (ex-) partners did not complete this scale.

**Table 7:** Frequency of impacts reported by men and (ex-) partners.

Number of Impacts	Men		(Ex-) Partners	
	Freq	%	Freq	%
(Partner) felt sadness	35	71.4	33	70.2
(Partner) felt angry/shocked	29	59.2	28	59.6
(Partner) lost respect for (men)	28	57.1	26	55.3
Made (partner) want to leave (men)	25	51.0	31	66.0
(Partner) felt anxious/panic/lost concentration	25	51.0	30	63.8
(Partner) stopped trusting (men)	24	49.0	30	63.8
(Partner) felt worthless or lost confidence	22	44.9	27	57.4
(Partner suffered) injuries such as bruises/scratches/minor cuts *	21	42.9	30	62.5
(Partner suffered) depression/sleeping problems *	20	40.8	28	59.6
(Partner) felt unable to cope	19	38.8	19	40.4
(Partner) had to be careful of what they said/did *	17	34.7	29	61.7
(Partner) felt isolated/stopped going out	12	24.5	18	38.3
(Partner) feared for their life	10	20.4	11	23.4
(Partner suffered) injuries needing help from doctor/hospital	8	16.3	9	19.1
(Partner) self-harmed/felt suicidal	8	16.3	13	27.7

* *p* < 0.05.

## Data Availability

Data sharing not applicable.
